# Bis(μ-l-arginine-κ^3^ *N* ^2^,*O*:*O*′)bis­(l-arginine-κ^2^ *N* ^2^,*O*)tetra-μ-chlorido-tetra­chlorido­tetra­copper(II)

**DOI:** 10.1107/S1600536813026780

**Published:** 2013-10-05

**Authors:** P. Arularasan, B.Sivakumar, G. Chakkaravarthi, R. Mohan

**Affiliations:** a Department of Physics, Presidency College, Chennai 600 005, India; b Department of Physics, CPCL Polytechnic College, Chennai 600 068, India

## Abstract

The title compound, [Cu_4_Cl_8_(C_6_H_14_N_4_O_2_)_4_], contains four mol­ecules in the asymmetric unit. In the mol­ecular structure, each of the four Cu^2+^ ions binds to three Cl atoms, one N atom and one O atom, resulting in distorted square-pyramidal coordination environments. The molecular structure is stabilized by weak C—H⋯O and N—H⋯Cl hydrogen bonds. The crystal structure exhibit weak inter­molecular N—H⋯O, C—H⋯O and N—H⋯Cl inter­actions, generating a three-dimensional network.

## Related literature

For general background of copper derivatives, see: Baran (2004 ▶); Sorenson (1976 ▶). For related structures, see: Ramaswamy *et al.* (2001 ▶); Sridhar *et al.* (2002 ▶); Sun *et al.* (2005 ▶); Wang *et al.* (2012 ▶).
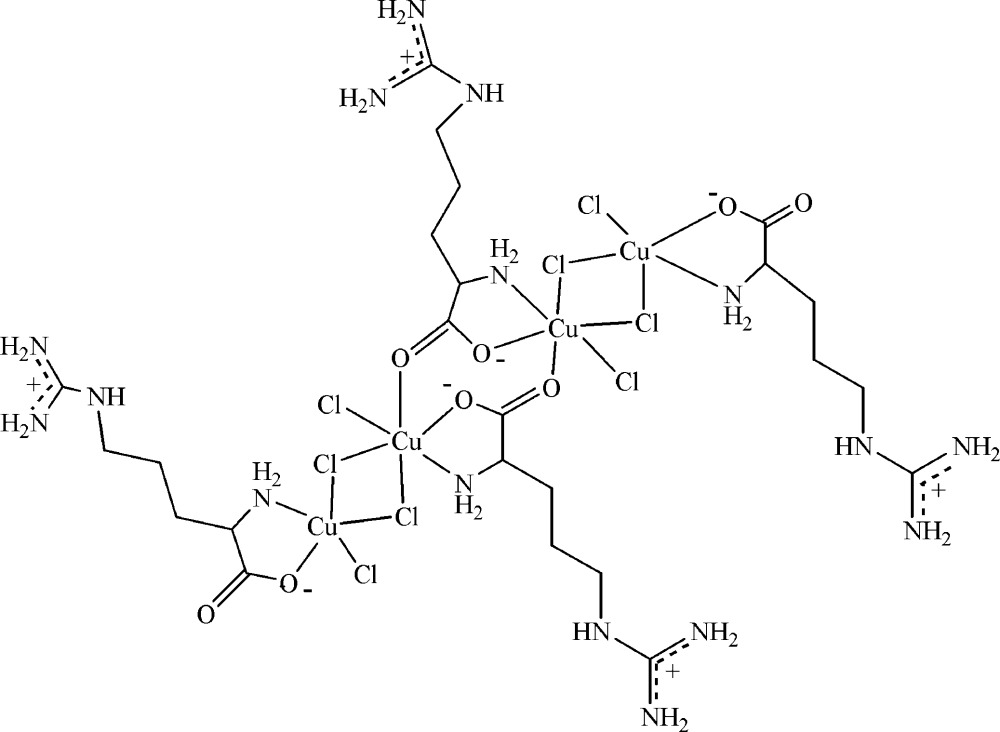


## Experimental

### 

#### Crystal data

[Cu_4_Cl_8_(C_6_H_14_N_4_O_2_)_4_]
*M* *_r_* = 1234.65
Monoclinic, 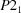

*a* = 11.9315 (8) Å
*b* = 12.8805 (10) Å
*c* = 15.3949 (13) Å
β = 99.271 (4)°
*V* = 2335.0 (3) Å^3^
*Z* = 2
Mo *K*α radiation
μ = 2.32 mm^−1^
*T* = 295 K
0.20 × 0.18 × 0.16 mm

#### Data collection

Bruker Kappa APEXII diffractometer
Absorption correction: multi-scan (*SADABS*; Sheldrick, 1996 ▶) *T*_min_ = 0.655, *T*_max_ = 0.708
16238 measured reflections
8443 independent reflections
6378 reflections with *I* > 2σ(*I*)
*R*_int_ = 0.053

#### Refinement

*R*[*F* ^2^ > 2σ(*F* ^2^)] = 0.047
*wR*(*F* ^2^) = 0.112
*S* = 0.98
8443 reflections
541 parameters
H-atom parameters constrained
Δρ_max_ = 0.98 e Å^−3^
Δρ_min_ = −0.54 e Å^−3^
Absolute structure: Flack (1983 ▶), 2876 Friedel pairs
Absolute structure parameter: −0.005 (13)

### 

Data collection: *APEX2* (Bruker, 2004 ▶); cell refinement: *SAINT* (Bruker, 2004 ▶); data reduction: *SAINT*; program(s) used to solve structure: *SHELXS97* (Sheldrick, 2008 ▶); program(s) used to refine structure: *SHELXL97* (Sheldrick, 2008 ▶); molecular graphics: *PLATON* (Spek, 2009 ▶); software used to prepare material for publication: *SHELXL97*.

## Supplementary Material

Crystal structure: contains datablock(s) I, global. DOI: 10.1107/S1600536813026780/rk2413sup1.cif

Structure factors: contains datablock(s) I. DOI: 10.1107/S1600536813026780/rk2413Isup2.hkl

Additional supplementary materials:  crystallographic information; 3D view; checkCIF report

## Figures and Tables

**Table 1:** Hydrogen-bond geometry (Å, °)

*D*—H⋯*A*	*D*—H	H⋯*A*	*D*⋯*A*	*D*—H⋯*A*
C9—H9*A*⋯O4	0.97	2.47	2.805 (7)	100
C22—H22*A*⋯O8	0.97	2.57	3.229 (8)	125
N13—H13*B*⋯Cl2	0.90	2.62	3.478 (5)	161
N1—H1*A*⋯Cl8	0.90	2.52	3.409 (5)	168
N5—H5*C*⋯Cl5	0.90	2.46	3.341 (5)	165
N2—H2*A*⋯O8^i^	0.86	2.02	2.857 (6)	166
N4—H4*F*⋯O7^i^	0.86	2.16	2.998 (7)	164
N10—H10⋯O4^ii^	0.86	1.95	2.791 (7)	167
N12—H12*B*⋯O3^ii^	0.86	2.17	2.975 (6)	156
N15—H15*C*⋯O1^iii^	0.86	2.02	2.873 (6)	171
N14—H14*A*⋯O2^iii^	0.86	2.01	2.873 (6)	176
N6—H6⋯O6^iv^	0.86	1.99	2.831 (6)	167
N7—H7*B*⋯O5^iv^	0.86	2.15	2.969 (7)	160
C20—H20⋯O3^v^	0.98	2.57	3.426 (7)	145
N3—H3*C*⋯Cl2^vi^	0.86	2.38	3.224 (7)	165
N7—H7*A*⋯Cl4^vi^	0.86	2.63	3.465 (6)	164
N8—H8*B*⋯Cl3^vi^	0.86	2.41	3.269 (6)	173
N11—H11*C*⋯Cl5^vii^	0.86	2.28	3.134 (6)	170
N12—H12*A*⋯Cl6^vii^	0.86	2.83	3.574 (6)	146
N16—H16*F*⋯Cl8^vii^	0.86	2.32	3.159 (6)	166
N11—H11*D*⋯Cl4^viii^	0.86	2.71	3.310 (5)	128
C17—H17*A*⋯Cl5^viii^	0.97	2.79	3.588 (6)	140
C23—H23*A*⋯Cl8^ix^	0.97	2.73	3.624 (7)	154
N16—H16*E*⋯Cl1^ix^	0.86	2.59	3.314 (5)	142
N8—H8*A*⋯Cl6^x^	0.86	2.69	3.300 (5)	130
N3—H3*D*⋯Cl7^xi^	0.86	2.70	3.329 (6)	131
N5—H5*D*⋯O8^xii^	0.90	2.33	3.041 (7)	136
C9—H9*B*⋯O7^xii^	0.97	2.60	3.460 (7)	148
N13—H13*A*⋯O4^v^	0.90	2.54	3.081 (7)	120

Symmetry codes: (i) 
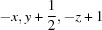
; (ii) 
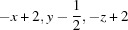
; (iii) 
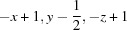
; (iv) 
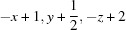
; (v) 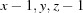; (vi) 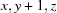; (vii) 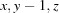; (viii) 
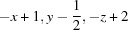
; (ix) 
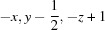
; (x) 
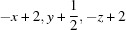
; (xi) 
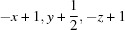
; (xii) 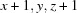.

**Fig. 1.**
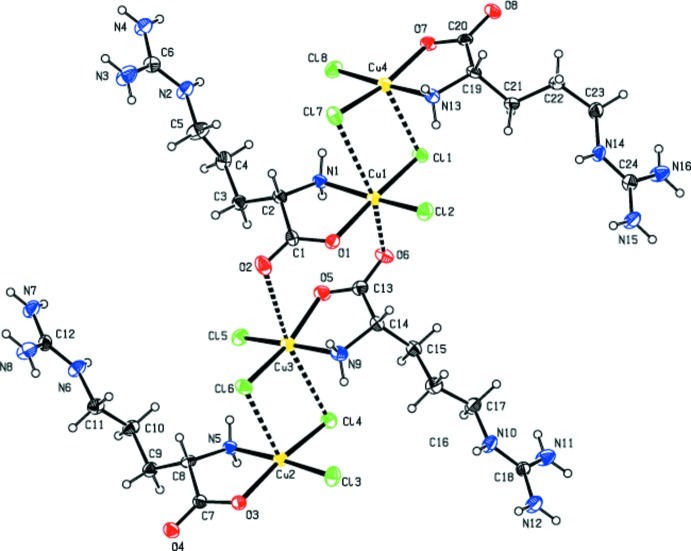
The molecular structure of title compound with the atom numbering scheme. Displacement ellipsoids are drawn at the 30% probability level. The H atoms are presented as a small spheres of arbitrary radius.

## supplementary crystallographic information

### 1. Comment

Copper complexes exhibit wide spectrum of effects such as anti–inflammatory, anti–cancer, anti–convulsant and anti–tumoral activities (Baran, 2004; Sorenson, 1976).

Four title molecules are present in the asymmetric unit. In the molecular structure a each copper atom binds to three Cl atoms and one N and one O atoms from organic ligand (Fig. 1), resulting in a distorted square-pyramidal coordination environment. In the molecular structure of title compound the bond distances of Cu—O lies in interval 1.954 (4)Å–1.966 (4)Å, Cu—N - 1.978 (5)Å–1.993 (5)Å and Cu—Cl - 2.2383 (17)Å–2.2867 (16)Å, and are in the normal range compared to the reported complexes (Sun *et al.*, 2005; Wang *et al.*, 2012). The geometric parameters of *L*–arginium moiety in title molecules are agree well with the reported similar structures (Ramaswamy *et al.*, 2001; Sridhar *et al.*, 2002).

The molecular structure is stabilized by weak C—H···O and N—H···Cl hydrogen bonds and the crystal structure is influenced by weak intermolecular N—H···O, C—H···O and N—H···Cl (Table 1) interactions to generate a three dimensional network.

### 2. Experimental

The title salt was synthesized from the starting materials of *L*–arginine (1.7420 g) and copper dichloride dihydrate (1.7048 g) taken in water solvent system. Single crystals suitable for X–ray diffraction were grown by slow evaporation technique at room temperature.

### 3. Refinement

The H atoms were positioned geometrically, with C—H = 0.97Å–0.98Å and N—H = 0.86Å–0.90Å, and allowed to ride on their parent atoms with *U*_iso_(H) = 1.2*U*_eq_(C, N).

### Crystal data

**Table:**

[Cu_4_Cl_8_(C_6_H_14_N_4_O_2_)_4_]	*F*(000) = 1256
*M**_r_* = 1234.65	*D*_x_ = 1.756 Mg m^−^^3^
Monoclinic, *P*2_1_	Mo *K*α radiation, λ = 0.71073 Å
Hall symbol: P 2yb	Cell parameters from 8444 reflections
*a* = 11.9315 (8) Å	θ = 1.3–27.6°
*b* = 12.8805 (10) Å	µ = 2.32 mm^−^^1^
*c* = 15.3949 (13) Å	*T* = 295 K
β = 99.271 (4)°	Block, blue
*V* = 2335.0 (3) Å^3^	0.20 × 0.18 × 0.16 mm
*Z* = 2	

### Data collection

**Table:**

Bruker Kappa APEXII diffractometer	8443 independent reflections
Radiation source: fine–focus sealed tube	6378 reflections with *I* > 2σ(*I*)
Graphite monochromator	*R*_int_ = 0.053
ω– and φ–scans	θ_max_ = 27.6°, θ_min_ = 1.3°
Absorption correction: multi-scan (*SADABS*; Sheldrick, 1996)	*h* = −11→15
*T*_min_ = 0.655, *T*_max_ = 0.708	*k* = −16→11
16238 measured reflections	*l* = −19→20

### Refinement

**Table:**

Refinement on *F*^2^	Secondary atom site location: difference Fourier map
Least-squares matrix: full	Hydrogen site location: inferred from neighbouring sites
*R*[*F*^2^ > 2σ(*F*^2^)] = 0.047	H-atom parameters constrained
*wR*(*F*^2^) = 0.112	*w* = 1/[σ^2^(*F*_o_^2^) + (0.0439*P*)^2^] where *P* = (*F*_o_^2^ + 2*F*_c_^2^)/3
*S* = 0.98	(Δ/σ)_max_ < 0.001
8443 reflections	Δρ_max_ = 0.98 e Å^−^^3^
541 parameters	Δρ_min_ = −0.54 e Å^−^^3^
0 restraints	Absolute structure: Flack (1983), 2876 Friedel pairs
Primary atom site location: structure-invariant direct methods	Absolute structure parameter: −0.005 (13)

### Special details

**Table:**

Geometry. All s.u.'s (except the s.u. in the dihedral angle between two l.s. planes) are estimated using the full covariance matrix. The cell s.u.'s are taken into account individually in the estimation of s.u.'s in distances, angles and torsion angles; correlations between s.u.'s in cell parameters are only used when they are defined by crystal symmetry. An approximate (isotropic) treatment of cell s.u.'s is used for estimating s.u.'s involving l.s. planes.
Refinement. Refinement of *F*^2^ against ALL reflections. The weighted *R*–factor *wR* and goodness of fit *S* are based on *F*^2^, conventional *R*–factors *R* are based on *F*, with *F* set to zero for negative *F*^2^. The threshold expression of *F*^2^ > σ(*F*^2^) is used only for calculating *R*–factors(gt) *etc*. and is not relevant to the choice of reflections for refinement. *R*–factors based on *F*^2^ are statistically about twice as large as those based on *F*, and *R*–factors based on ALL data will be even larger.

### Fractional atomic coordinates and isotropic or equivalent isotropic displacement parameters (Å^2^)

**Table:**

	*x*	*y*	*z*	*U*_iso_*/*U*_eq_	
C1	0.5534 (5)	0.3535 (5)	0.6619 (4)	0.0314 (14)	
C2	0.4492 (4)	0.4237 (5)	0.6536 (4)	0.0293 (13)	
H2	0.4449	0.4610	0.5977	0.035*	
C3	0.4546 (5)	0.5055 (5)	0.7253 (4)	0.0338 (14)	
H3A	0.4540	0.4707	0.7811	0.041*	
H3B	0.5262	0.5422	0.7293	0.041*	
C4	0.3591 (5)	0.5841 (5)	0.7117 (5)	0.0392 (16)	
H4A	0.2876	0.5482	0.6939	0.047*	
H4B	0.3554	0.6189	0.7670	0.047*	
C5	0.3742 (6)	0.6641 (7)	0.6432 (5)	0.054 (2)	
H5A	0.4432	0.7031	0.6624	0.064*	
H5B	0.3819	0.6295	0.5885	0.064*	
C6	0.2832 (5)	0.8286 (5)	0.5938 (4)	0.0360 (16)	
C7	1.0311 (5)	0.2921 (5)	1.1448 (4)	0.0287 (13)	
C8	0.9244 (4)	0.3595 (5)	1.1414 (4)	0.0292 (14)	
H8	0.9175	0.4005	1.0871	0.035*	
C9	0.9305 (5)	0.4355 (5)	1.2162 (4)	0.0360 (15)	
H9A	1.0044	0.4686	1.2246	0.043*	
H9B	0.9243	0.3974	1.2696	0.043*	
C10	0.8397 (5)	0.5195 (5)	1.2036 (4)	0.0383 (15)	
H10A	0.7701	0.4908	1.1711	0.046*	
H10B	0.8242	0.5416	1.2607	0.046*	
C11	0.8748 (5)	0.6123 (5)	1.1547 (5)	0.0391 (16)	
H11A	0.9466	0.6390	1.1850	0.047*	
H11B	0.8850	0.5918	1.0959	0.047*	
C12	0.7940 (5)	0.7836 (5)	1.1116 (4)	0.0350 (15)	
C13	0.4773 (5)	0.1676 (5)	0.7897 (4)	0.0327 (14)	
C14	0.5726 (5)	0.0932 (5)	0.7828 (4)	0.0309 (14)	
H14	0.5749	0.0782	0.7207	0.037*	
C15	0.5476 (5)	−0.0066 (5)	0.8300 (4)	0.0363 (15)	
H15A	0.5420	0.0098	0.8906	0.044*	
H15B	0.4746	−0.0336	0.8024	0.044*	
C16	0.6358 (5)	−0.0892 (6)	0.8288 (5)	0.0426 (17)	
H16A	0.7073	−0.0656	0.8618	0.051*	
H16B	0.6468	−0.1015	0.7686	0.051*	
C17	0.6010 (5)	−0.1888 (5)	0.8680 (5)	0.0414 (16)	
H17A	0.5829	−0.1753	0.9262	0.050*	
H17B	0.5335	−0.2161	0.8317	0.050*	
C18	0.6854 (5)	−0.3587 (5)	0.9072 (4)	0.0328 (15)	
C19	0.0012 (5)	0.1880 (5)	0.3325 (4)	0.0293 (14)	
C20	0.1079 (4)	0.1333 (5)	0.3163 (4)	0.0283 (14)	
H20	0.1057	0.1296	0.2524	0.034*	
C21	0.1155 (5)	0.0228 (5)	0.3515 (4)	0.0331 (14)	
H21A	0.0851	0.0204	0.4062	0.040*	
H21B	0.1945	0.0018	0.3638	0.040*	
C22	0.0497 (5)	−0.0528 (5)	0.2857 (4)	0.0392 (15)	
H22A	−0.0261	−0.0256	0.2668	0.047*	
H22B	0.0870	−0.0572	0.2343	0.047*	
C23	0.0406 (5)	−0.1608 (5)	0.3228 (5)	0.0439 (17)	
H23A	0.0068	−0.1564	0.3759	0.053*	
H23B	−0.0091	−0.2025	0.2804	0.053*	
C24	0.1617 (5)	−0.3054 (5)	0.3774 (4)	0.0347 (16)	
N1	0.3473 (4)	0.3563 (4)	0.6459 (3)	0.0300 (12)	
H1A	0.2879	0.3883	0.6132	0.036*	
H1B	0.3296	0.3443	0.6996	0.036*	
N2	0.2789 (4)	0.7344 (4)	0.6281 (4)	0.0413 (14)	
H2A	0.2156	0.7142	0.6423	0.050*	
N3	0.3784 (5)	0.8669 (5)	0.5730 (5)	0.064 (2)	
H3C	0.3790	0.9282	0.5508	0.077*	
H3D	0.4396	0.8306	0.5817	0.077*	
N4	0.1902 (4)	0.8861 (5)	0.5797 (4)	0.0452 (15)	
H4E	0.1923	0.9472	0.5574	0.054*	
H4F	0.1276	0.8625	0.5928	0.054*	
N5	0.8283 (4)	0.2870 (4)	1.1305 (3)	0.0307 (12)	
H5C	0.7654	0.3191	1.1030	0.037*	
H5D	0.8150	0.2658	1.1836	0.037*	
N6	0.7884 (4)	0.6924 (4)	1.1496 (4)	0.0381 (14)	
H6	0.7292	0.6795	1.1731	0.046*	
N7	0.7083 (4)	0.8488 (4)	1.1103 (4)	0.0458 (15)	
H7A	0.7106	0.9089	1.0863	0.055*	
H7B	0.6501	0.8310	1.1333	0.055*	
N8	0.8835 (4)	0.8100 (5)	1.0764 (4)	0.0498 (16)	
H8A	0.9394	0.7675	1.0781	0.060*	
H8B	0.8861	0.8697	1.0518	0.060*	
N9	0.6816 (4)	0.1427 (4)	0.8237 (3)	0.0362 (13)	
H9C	0.7201	0.1634	0.7811	0.043*	
H9D	0.7245	0.0957	0.8573	0.043*	
N10	0.6912 (4)	−0.2646 (4)	0.8742 (4)	0.0408 (14)	
H10	0.7525	−0.2473	0.8552	0.049*	
N11	0.5912 (4)	−0.3904 (5)	0.9308 (4)	0.0517 (17)	
H11C	0.5866	−0.4523	0.9510	0.062*	
H11D	0.5336	−0.3495	0.9263	0.062*	
N12	0.7755 (4)	−0.4191 (4)	0.9132 (4)	0.0466 (15)	
H12A	0.7730	−0.4813	0.9331	0.056*	
H12B	0.8368	−0.3963	0.8972	0.056*	
N13	0.2093 (4)	0.1961 (4)	0.3535 (3)	0.0330 (13)	
H13A	0.2418	0.2219	0.3093	0.040*	
H13B	0.2604	0.1549	0.3864	0.040*	
N14	0.1512 (4)	−0.2119 (4)	0.3432 (3)	0.0337 (13)	
H14A	0.2108	−0.1799	0.3326	0.040*	
N15	0.2627 (4)	−0.3505 (5)	0.3923 (4)	0.0483 (16)	
H15C	0.3212	−0.3184	0.3796	0.058*	
H15D	0.2699	−0.4117	0.4147	0.058*	
N16	0.0716 (4)	−0.3562 (5)	0.3939 (4)	0.0506 (16)	
H16E	0.0055	−0.3281	0.3823	0.061*	
H16F	0.0790	−0.4175	0.4163	0.061*	
O1	0.5368 (3)	0.2637 (3)	0.6296 (3)	0.0353 (10)	
O2	0.6472 (3)	0.3859 (4)	0.6976 (4)	0.0527 (13)	
O3	1.0198 (3)	0.2071 (3)	1.1024 (3)	0.0332 (10)	
O4	1.1221 (3)	0.3230 (4)	1.1867 (3)	0.0389 (11)	
O5	0.4957 (3)	0.2445 (4)	0.8428 (3)	0.0406 (11)	
O6	0.3819 (3)	0.1501 (4)	0.7468 (3)	0.0400 (11)	
O7	0.0126 (3)	0.2638 (3)	0.3875 (3)	0.0335 (10)	
O8	−0.0918 (3)	0.1595 (4)	0.2951 (3)	0.0400 (11)	
Cl1	0.19231 (11)	0.17251 (13)	0.56835 (10)	0.0343 (4)	
Cl2	0.43100 (13)	0.09564 (13)	0.50464 (12)	0.0419 (4)	
Cl3	0.91855 (14)	0.03289 (13)	0.98284 (12)	0.0456 (4)	
Cl4	0.67991 (12)	0.10661 (13)	1.04626 (12)	0.0381 (4)	
Cl5	0.60355 (14)	0.37797 (13)	0.99408 (12)	0.0419 (4)	
Cl6	0.83827 (11)	0.30953 (14)	0.91470 (11)	0.0384 (4)	
Cl7	0.34317 (13)	0.38180 (14)	0.42749 (12)	0.0440 (4)	
Cl8	0.11091 (14)	0.44053 (13)	0.50966 (12)	0.0435 (4)	
Cu1	0.37830 (6)	0.22228 (5)	0.58961 (5)	0.03038 (19)	
Cu2	0.86295 (6)	0.16497 (6)	1.06027 (5)	0.03005 (18)	
Cu3	0.65413 (6)	0.26337 (6)	0.89680 (5)	0.0352 (2)	
Cu4	0.16845 (6)	0.31178 (6)	0.42699 (5)	0.03190 (19)	

### Atomic displacement parameters (Å^2^)

**Table:**

	*U*^11^	*U*^22^	*U*^33^	*U*^12^	*U*^13^	*U*^23^
C1	0.029 (3)	0.031 (4)	0.034 (4)	−0.006 (3)	0.005 (3)	−0.004 (3)
C2	0.029 (3)	0.033 (4)	0.026 (3)	−0.003 (3)	0.003 (2)	−0.001 (3)
C3	0.032 (3)	0.035 (4)	0.033 (4)	0.001 (3)	0.004 (3)	−0.002 (3)
C4	0.041 (4)	0.036 (4)	0.045 (4)	0.001 (3)	0.020 (3)	−0.008 (3)
C5	0.041 (4)	0.060 (5)	0.066 (5)	0.018 (4)	0.026 (4)	0.020 (4)
C6	0.035 (3)	0.042 (4)	0.033 (4)	0.000 (3)	0.012 (3)	0.004 (3)
C7	0.027 (3)	0.030 (4)	0.029 (3)	−0.001 (3)	0.004 (2)	0.002 (3)
C8	0.027 (3)	0.030 (3)	0.031 (3)	0.007 (3)	0.005 (2)	0.003 (3)
C9	0.044 (4)	0.029 (4)	0.033 (4)	0.007 (3)	0.003 (3)	−0.003 (3)
C10	0.044 (4)	0.035 (4)	0.036 (4)	0.009 (3)	0.009 (3)	0.001 (3)
C11	0.034 (3)	0.038 (4)	0.047 (4)	0.010 (3)	0.010 (3)	−0.002 (3)
C12	0.036 (3)	0.027 (4)	0.042 (4)	−0.001 (3)	0.005 (3)	−0.007 (3)
C13	0.030 (3)	0.035 (4)	0.035 (4)	−0.002 (3)	0.008 (3)	0.008 (3)
C14	0.029 (3)	0.034 (4)	0.030 (3)	−0.003 (3)	0.004 (2)	−0.002 (3)
C15	0.031 (3)	0.041 (4)	0.036 (4)	−0.004 (3)	0.004 (3)	0.000 (3)
C16	0.038 (4)	0.044 (4)	0.047 (4)	0.001 (3)	0.010 (3)	0.005 (3)
C17	0.036 (3)	0.033 (4)	0.056 (5)	0.002 (3)	0.010 (3)	0.005 (4)
C18	0.027 (3)	0.028 (4)	0.042 (4)	0.003 (3)	0.003 (3)	−0.003 (3)
C19	0.030 (3)	0.030 (4)	0.026 (3)	0.003 (3)	−0.001 (3)	0.009 (3)
C20	0.024 (3)	0.037 (4)	0.023 (3)	−0.002 (3)	−0.001 (2)	0.002 (3)
C21	0.029 (3)	0.034 (4)	0.034 (4)	0.005 (3)	−0.002 (2)	0.001 (3)
C22	0.042 (4)	0.036 (4)	0.036 (4)	0.007 (3)	−0.008 (3)	−0.003 (3)
C23	0.032 (3)	0.032 (4)	0.066 (5)	0.002 (3)	0.002 (3)	−0.006 (3)
C24	0.031 (3)	0.035 (4)	0.040 (4)	−0.001 (3)	0.013 (3)	0.000 (3)
N1	0.020 (2)	0.035 (3)	0.034 (3)	0.002 (2)	0.002 (2)	−0.001 (2)
N2	0.032 (3)	0.032 (3)	0.064 (4)	0.004 (3)	0.022 (3)	0.008 (3)
N3	0.047 (4)	0.050 (4)	0.099 (6)	0.001 (3)	0.025 (4)	0.029 (4)
N4	0.043 (3)	0.038 (3)	0.056 (4)	0.005 (3)	0.014 (3)	0.009 (3)
N5	0.023 (2)	0.034 (3)	0.034 (3)	0.003 (2)	0.002 (2)	−0.001 (2)
N6	0.032 (3)	0.034 (3)	0.053 (4)	0.010 (2)	0.020 (2)	0.007 (3)
N7	0.039 (3)	0.032 (3)	0.068 (4)	0.000 (3)	0.013 (3)	0.013 (3)
N8	0.035 (3)	0.050 (4)	0.067 (4)	0.005 (3)	0.019 (3)	0.020 (3)
N9	0.024 (2)	0.046 (3)	0.039 (3)	0.002 (2)	0.006 (2)	0.003 (3)
N10	0.035 (3)	0.028 (3)	0.062 (4)	0.001 (3)	0.015 (3)	0.009 (3)
N11	0.035 (3)	0.047 (4)	0.075 (5)	0.004 (3)	0.014 (3)	0.025 (3)
N12	0.042 (3)	0.034 (3)	0.065 (4)	0.004 (3)	0.012 (3)	0.012 (3)
N13	0.027 (2)	0.038 (3)	0.034 (3)	−0.001 (2)	0.003 (2)	0.000 (2)
N14	0.031 (3)	0.024 (3)	0.047 (3)	−0.004 (2)	0.010 (2)	0.003 (2)
N15	0.038 (3)	0.034 (3)	0.074 (4)	0.004 (3)	0.013 (3)	0.016 (3)
N16	0.036 (3)	0.050 (4)	0.067 (4)	0.003 (3)	0.012 (3)	0.027 (3)
O1	0.0183 (19)	0.029 (2)	0.058 (3)	0.0028 (19)	0.0057 (18)	0.004 (2)
O2	0.027 (2)	0.052 (3)	0.077 (4)	−0.001 (2)	0.003 (2)	−0.021 (3)
O3	0.024 (2)	0.032 (3)	0.043 (3)	0.0044 (19)	0.0040 (18)	−0.004 (2)
O4	0.030 (2)	0.043 (3)	0.043 (3)	−0.002 (2)	0.0033 (19)	−0.002 (2)
O5	0.028 (2)	0.043 (3)	0.049 (3)	0.008 (2)	0.0003 (19)	−0.006 (2)
O6	0.028 (2)	0.056 (3)	0.034 (3)	−0.001 (2)	0.0004 (18)	0.003 (2)
O7	0.0225 (19)	0.040 (3)	0.037 (3)	0.0081 (19)	0.0036 (17)	−0.004 (2)
O8	0.022 (2)	0.045 (3)	0.051 (3)	0.000 (2)	−0.0017 (19)	−0.004 (2)
Cl1	0.0237 (7)	0.0420 (9)	0.0365 (9)	−0.0043 (7)	0.0025 (6)	0.0038 (8)
Cl2	0.0402 (9)	0.0365 (10)	0.0507 (11)	0.0044 (7)	0.0128 (8)	−0.0058 (8)
Cl3	0.0485 (9)	0.0363 (10)	0.0555 (11)	0.0023 (8)	0.0191 (8)	−0.0112 (8)
Cl4	0.0292 (7)	0.0412 (10)	0.0442 (10)	−0.0031 (7)	0.0073 (7)	0.0018 (8)
Cl5	0.0430 (9)	0.0351 (9)	0.0481 (10)	0.0085 (8)	0.0095 (7)	−0.0014 (8)
Cl6	0.0277 (7)	0.0470 (10)	0.0399 (9)	−0.0052 (7)	0.0037 (6)	0.0059 (8)
Cl7	0.0361 (8)	0.0503 (11)	0.0461 (10)	−0.0131 (8)	0.0081 (7)	−0.0052 (8)
Cl8	0.0498 (10)	0.0377 (10)	0.0441 (10)	0.0107 (8)	0.0107 (8)	−0.0012 (8)
Cu1	0.0227 (3)	0.0310 (4)	0.0365 (5)	0.0014 (3)	0.0020 (3)	−0.0018 (4)
Cu2	0.0260 (4)	0.0320 (4)	0.0321 (4)	0.0026 (3)	0.0047 (3)	−0.0005 (4)
Cu3	0.0248 (4)	0.0364 (5)	0.0434 (5)	0.0027 (3)	0.0026 (3)	−0.0038 (4)
Cu4	0.0271 (4)	0.0336 (4)	0.0338 (4)	0.0020 (3)	0.0015 (3)	−0.0009 (4)

### Geometric parameters (Å, º)

**Table:**

C1—O2	1.238 (7)	C20—C21	1.521 (9)
C1—O1	1.263 (7)	C20—H20	0.9800
C1—C2	1.526 (8)	C21—C22	1.528 (8)
C2—N1	1.483 (7)	C21—H21A	0.9700
C2—C3	1.518 (8)	C21—H21B	0.9700
C2—H2	0.9800	C22—C23	1.515 (9)
C3—C4	1.515 (8)	C22—H22A	0.9700
C3—H3A	0.9700	C22—H22B	0.9700
C3—H3B	0.9700	C23—N14	1.462 (8)
C4—C5	1.506 (10)	C23—H23A	0.9700
C4—H4A	0.9700	C23—H23B	0.9700
C4—H4B	0.9700	C24—N14	1.313 (8)
C5—N2	1.443 (8)	C24—N16	1.317 (8)
C5—H5A	0.9700	C24—N15	1.324 (8)
C5—H5B	0.9700	N1—Cu1	1.993 (5)
C6—N4	1.323 (8)	N1—H1A	0.9000
C6—N3	1.323 (8)	N1—H1B	0.9000
C6—N2	1.328 (8)	N2—H2A	0.8600
C7—O4	1.236 (7)	N3—H3C	0.8600
C7—O3	1.270 (7)	N3—H3D	0.8600
C7—C8	1.535 (8)	N4—H4E	0.8600
C8—N5	1.468 (7)	N4—H4F	0.8600
C8—C9	1.504 (8)	N5—Cu2	1.988 (5)
C8—H8	0.9800	N5—H5C	0.9000
C9—C10	1.521 (8)	N5—H5D	0.9000
C9—H9A	0.9700	N6—H6	0.8600
C9—H9B	0.9700	N7—H7A	0.8600
C10—C11	1.507 (9)	N7—H7B	0.8600
C10—H10A	0.9700	N8—H8A	0.8600
C10—H10B	0.9700	N8—H8B	0.8600
C11—N6	1.453 (7)	N9—Cu3	1.978 (5)
C11—H11A	0.9700	N9—H9C	0.9000
C11—H11B	0.9700	N9—H9D	0.9000
C12—N8	1.317 (8)	N10—H10	0.8600
C12—N6	1.320 (8)	N11—H11C	0.8600
C12—N7	1.321 (8)	N11—H11D	0.8600
C13—O6	1.241 (7)	N12—H12A	0.8600
C13—O5	1.281 (8)	N12—H12B	0.8600
C13—C14	1.504 (8)	N13—Cu4	1.978 (5)
C14—N9	1.493 (7)	N13—H13A	0.9000
C14—C15	1.530 (9)	N13—H13B	0.9000
C14—H14	0.9800	N14—H14A	0.8600
C15—C16	1.498 (9)	N15—H15C	0.8600
C15—H15A	0.9700	N15—H15D	0.8600
C15—H15B	0.9700	N16—H16E	0.8600
C16—C17	1.506 (9)	N16—H16F	0.8600
C16—H16A	0.9700	O1—Cu1	1.966 (4)
C16—H16B	0.9700	O3—Cu2	1.956 (4)
C17—N10	1.444 (8)	O5—Cu3	1.954 (4)
C17—H17A	0.9700	O7—Cu4	1.960 (4)
C17—H17B	0.9700	Cl1—Cu1	2.2823 (15)
C18—N11	1.301 (8)	Cl2—Cu1	2.2433 (18)
C18—N12	1.319 (8)	Cl3—Cu2	2.2383 (17)
C18—N10	1.320 (8)	Cl4—Cu2	2.2867 (16)
C19—O8	1.221 (7)	Cl5—Cu3	2.2532 (18)
C19—O7	1.285 (7)	Cl6—Cu3	2.2498 (15)
C19—C20	1.511 (8)	Cl7—Cu4	2.2704 (16)
C20—N13	1.491 (7)	Cl8—Cu4	2.2633 (18)
			
O2—C1—O1	123.8 (6)	H21A—C21—H21B	108.0
O2—C1—C2	120.3 (6)	C23—C22—C21	113.4 (5)
O1—C1—C2	115.9 (5)	C23—C22—H22A	108.9
N1—C2—C3	113.8 (4)	C21—C22—H22A	108.9
N1—C2—C1	107.8 (5)	C23—C22—H22B	108.9
C3—C2—C1	114.3 (5)	C21—C22—H22B	108.9
N1—C2—H2	106.8	H22A—C22—H22B	107.7
C3—C2—H2	106.8	N14—C23—C22	112.1 (5)
C1—C2—H2	106.8	N14—C23—H23A	109.2
C4—C3—C2	114.9 (5)	C22—C23—H23A	109.2
C4—C3—H3A	108.6	N14—C23—H23B	109.2
C2—C3—H3A	108.6	C22—C23—H23B	109.2
C4—C3—H3B	108.6	H23A—C23—H23B	107.9
C2—C3—H3B	108.6	N14—C24—N16	120.4 (6)
H3A—C3—H3B	107.5	N14—C24—N15	119.8 (6)
C5—C4—C3	112.5 (5)	N16—C24—N15	119.8 (6)
C5—C4—H4A	109.1	C2—N1—Cu1	109.6 (3)
C3—C4—H4A	109.1	C2—N1—H1A	109.8
C5—C4—H4B	109.1	Cu1—N1—H1A	109.8
C3—C4—H4B	109.1	C2—N1—H1B	109.8
H4A—C4—H4B	107.8	Cu1—N1—H1B	109.8
N2—C5—C4	111.2 (5)	H1A—N1—H1B	108.2
N2—C5—H5A	109.4	C6—N2—C5	123.9 (6)
C4—C5—H5A	109.4	C6—N2—H2A	118.1
N2—C5—H5B	109.4	C5—N2—H2A	118.1
C4—C5—H5B	109.4	C6—N3—H3C	120.0
H5A—C5—H5B	108.0	C6—N3—H3D	120.0
N4—C6—N3	118.8 (6)	H3C—N3—H3D	120.0
N4—C6—N2	119.4 (6)	C6—N4—H4E	120.0
N3—C6—N2	121.8 (6)	C6—N4—H4F	120.0
O4—C7—O3	123.9 (6)	H4E—N4—H4F	120.0
O4—C7—C8	119.3 (6)	C8—N5—Cu2	109.7 (3)
O3—C7—C8	116.8 (5)	C8—N5—H5C	109.7
N5—C8—C9	116.2 (5)	Cu2—N5—H5C	109.7
N5—C8—C7	105.8 (5)	C8—N5—H5D	109.7
C9—C8—C7	113.9 (5)	Cu2—N5—H5D	109.7
N5—C8—H8	106.8	H5C—N5—H5D	108.2
C9—C8—H8	106.8	C12—N6—C11	124.8 (5)
C7—C8—H8	106.8	C12—N6—H6	117.6
C8—C9—C10	114.8 (5)	C11—N6—H6	117.6
C8—C9—H9A	108.6	C12—N7—H7A	120.0
C10—C9—H9A	108.6	C12—N7—H7B	120.0
C8—C9—H9B	108.6	H7A—N7—H7B	120.0
C10—C9—H9B	108.6	C12—N8—H8A	120.0
H9A—C9—H9B	107.5	C12—N8—H8B	120.0
C11—C10—C9	112.2 (5)	H8A—N8—H8B	120.0
C11—C10—H10A	109.2	C14—N9—Cu3	111.3 (3)
C9—C10—H10A	109.2	C14—N9—H9C	109.4
C11—C10—H10B	109.2	Cu3—N9—H9C	109.4
C9—C10—H10B	109.2	C14—N9—H9D	109.4
H10A—C10—H10B	107.9	Cu3—N9—H9D	109.4
N6—C11—C10	109.8 (5)	H9C—N9—H9D	108.0
N6—C11—H11A	109.7	C18—N10—C17	124.1 (5)
C10—C11—H11A	109.7	C18—N10—H10	117.9
N6—C11—H11B	109.7	C17—N10—H10	117.9
C10—C11—H11B	109.7	C18—N11—H11C	120.0
H11A—C11—H11B	108.2	C18—N11—H11D	120.0
N8—C12—N6	121.0 (6)	H11C—N11—H11D	120.0
N8—C12—N7	120.5 (6)	C18—N12—H12A	120.0
N6—C12—N7	118.5 (6)	C18—N12—H12B	120.0
O6—C13—O5	121.9 (6)	H12A—N12—H12B	120.0
O6—C13—C14	119.1 (6)	C20—N13—Cu4	111.7 (3)
O5—C13—C14	118.9 (5)	C20—N13—H13A	109.3
N9—C14—C13	108.4 (5)	Cu4—N13—H13A	109.3
N9—C14—C15	112.4 (5)	C20—N13—H13B	109.3
C13—C14—C15	107.1 (5)	Cu4—N13—H13B	109.3
N9—C14—H14	109.6	H13A—N13—H13B	107.9
C13—C14—H14	109.6	C24—N14—C23	121.6 (5)
C15—C14—H14	109.6	C24—N14—H14A	119.2
C16—C15—C14	113.5 (5)	C23—N14—H14A	119.2
C16—C15—H15A	108.9	C24—N15—H15C	120.0
C14—C15—H15A	108.9	C24—N15—H15D	120.0
C16—C15—H15B	108.9	H15C—N15—H15D	120.0
C14—C15—H15B	108.9	C24—N16—H16E	120.0
H15A—C15—H15B	107.7	C24—N16—H16F	120.0
C15—C16—C17	111.1 (5)	H16E—N16—H16F	120.0
C15—C16—H16A	109.4	C1—O1—Cu1	117.0 (4)
C17—C16—H16A	109.4	C7—O3—Cu2	115.2 (4)
C15—C16—H16B	109.4	C13—O5—Cu3	115.0 (4)
C17—C16—H16B	109.4	C19—O7—Cu4	116.0 (3)
H16A—C16—H16B	108.0	O1—Cu1—N1	82.28 (18)
N10—C17—C16	110.6 (5)	O1—Cu1—Cl2	91.94 (13)
N10—C17—H17A	109.5	N1—Cu1—Cl2	166.55 (15)
C16—C17—H17A	109.5	O1—Cu1—Cl1	170.14 (14)
N10—C17—H17B	109.5	N1—Cu1—Cl1	93.52 (13)
C16—C17—H17B	109.5	Cl2—Cu1—Cl1	93.99 (6)
H17A—C17—H17B	108.1	O3—Cu2—N5	82.67 (18)
N11—C18—N12	121.8 (6)	O3—Cu2—Cl3	92.15 (13)
N11—C18—N10	119.8 (6)	N5—Cu2—Cl3	174.72 (14)
N12—C18—N10	118.4 (6)	O3—Cu2—Cl4	166.08 (14)
O8—C19—O7	122.1 (5)	N5—Cu2—Cl4	91.82 (14)
O8—C19—C20	120.5 (6)	Cl3—Cu2—Cl4	93.46 (7)
O7—C19—C20	117.4 (5)	O5—Cu3—N9	84.12 (19)
N13—C20—C19	109.7 (5)	O5—Cu3—Cl6	160.68 (15)
N13—C20—C21	111.9 (5)	N9—Cu3—Cl6	91.69 (14)
C19—C20—C21	112.4 (5)	O5—Cu3—Cl5	91.14 (14)
N13—C20—H20	107.6	N9—Cu3—Cl5	168.54 (16)
C19—C20—H20	107.6	Cl6—Cu3—Cl5	96.10 (7)
C21—C20—H20	107.6	O7—Cu4—N13	83.92 (18)
C20—C21—C22	111.4 (5)	O7—Cu4—Cl8	92.69 (13)
C20—C21—H21A	109.3	N13—Cu4—Cl8	176.58 (14)
C22—C21—H21A	109.3	O7—Cu4—Cl7	161.90 (13)
C20—C21—H21B	109.3	N13—Cu4—Cl7	89.44 (14)
C22—C21—H21B	109.3	Cl8—Cu4—Cl7	93.93 (7)
			
O2—C1—C2—N1	−155.8 (6)	C10—C11—N6—C12	179.5 (6)
O1—C1—C2—N1	25.0 (7)	C13—C14—N9—Cu3	15.5 (6)
O2—C1—C2—C3	−28.3 (8)	C15—C14—N9—Cu3	−102.7 (5)
O1—C1—C2—C3	152.5 (6)	N11—C18—N10—C17	4.9 (11)
N1—C2—C3—C4	−63.0 (7)	N12—C18—N10—C17	−176.8 (6)
C1—C2—C3—C4	172.6 (5)	C16—C17—N10—C18	179.0 (7)
C2—C3—C4—C5	−76.2 (7)	C19—C20—N13—Cu4	10.0 (6)
C3—C4—C5—N2	176.8 (6)	C21—C20—N13—Cu4	−115.4 (5)
O4—C7—C8—N5	−151.3 (5)	N16—C24—N14—C23	0.3 (10)
O3—C7—C8—N5	29.7 (7)	N15—C24—N14—C23	177.8 (6)
O4—C7—C8—C9	−22.5 (8)	C22—C23—N14—C24	178.8 (6)
O3—C7—C8—C9	158.5 (5)	O2—C1—O1—Cu1	172.4 (5)
N5—C8—C9—C10	−70.3 (7)	C2—C1—O1—Cu1	−8.4 (7)
C7—C8—C9—C10	166.4 (5)	O4—C7—O3—Cu2	169.3 (5)
C8—C9—C10—C11	−87.2 (7)	C8—C7—O3—Cu2	−11.8 (7)
C9—C10—C11—N6	−176.3 (5)	O6—C13—O5—Cu3	−175.7 (4)
O6—C13—C14—N9	167.7 (5)	C14—C13—O5—Cu3	7.6 (7)
O5—C13—C14—N9	−15.5 (8)	O8—C19—O7—Cu4	−171.0 (5)
O6—C13—C14—C15	−70.8 (7)	C20—C19—O7—Cu4	9.8 (7)
O5—C13—C14—C15	106.1 (6)	C1—O1—Cu1—N1	−7.2 (5)
N9—C14—C15—C16	−61.0 (7)	C1—O1—Cu1—Cl2	160.6 (4)
C13—C14—C15—C16	180.0 (5)	C2—N1—Cu1—O1	20.6 (4)
C14—C15—C16—C17	−174.3 (6)	C2—N1—Cu1—Cl2	−44.5 (9)
C15—C16—C17—N10	−174.5 (6)	C2—N1—Cu1—Cl1	−168.3 (4)
O8—C19—C20—N13	167.8 (5)	C7—O3—Cu2—N5	−6.4 (4)
O7—C19—C20—N13	−13.1 (7)	C7—O3—Cu2—Cl3	172.6 (4)
O8—C19—C20—C21	−67.2 (7)	C7—O3—Cu2—Cl4	−73.7 (7)
O7—C19—C20—C21	112.0 (6)	C8—N5—Cu2—O3	23.0 (4)
N13—C20—C21—C22	−152.0 (5)	C8—N5—Cu2—Cl4	−169.9 (4)
C19—C20—C21—C22	84.1 (6)	C13—O5—Cu3—N9	1.7 (4)
C20—C21—C22—C23	−171.7 (5)	C13—O5—Cu3—Cl6	80.0 (6)
C21—C22—C23—N14	−65.3 (7)	C13—O5—Cu3—Cl5	−167.8 (4)
C3—C2—N1—Cu1	−156.7 (4)	C14—N9—Cu3—O5	−10.2 (4)
C1—C2—N1—Cu1	−28.9 (5)	C14—N9—Cu3—Cl6	−171.3 (4)
N4—C6—N2—C5	177.7 (7)	C14—N9—Cu3—Cl5	55.8 (10)
N3—C6—N2—C5	−2.3 (11)	C19—O7—Cu4—N13	−2.9 (4)
C4—C5—N2—C6	158.3 (7)	C19—O7—Cu4—Cl8	177.6 (4)
C9—C8—N5—Cu2	−159.9 (4)	C19—O7—Cu4—Cl7	66.2 (6)
C7—C8—N5—Cu2	−32.4 (5)	C20—N13—Cu4—O7	−4.6 (4)
N8—C12—N6—C11	−1.9 (11)	C20—N13—Cu4—Cl7	−167.7 (4)
N7—C12—N6—C11	178.8 (6)		

### Hydrogen-bond geometry (Å, º)

**Table:**

*D*—H···*A*	*D*—H	H···*A*	*D*···*A*	*D*—H···*A*
C9—H9*A*···O4	0.97	2.47	2.805 (7)	100
C22—H22*A*···O8	0.97	2.57	3.229 (8)	125
N13—H13*B*···Cl2	0.90	2.62	3.478 (5)	161
N1—H1*A*···Cl8	0.90	2.52	3.409 (5)	168
N5—H5*C*···Cl5	0.90	2.46	3.341 (5)	165
N2—H2*A*···O8^i^	0.86	2.02	2.857 (6)	166
N4—H4*F*···O7^i^	0.86	2.16	2.998 (7)	164
N10—H10···O4^ii^	0.86	1.95	2.791 (7)	167
N12—H12*B*···O3^ii^	0.86	2.17	2.975 (6)	156
N15—H15*C*···O1^iii^	0.86	2.02	2.873 (6)	171
N14—H14*A*···O2^iii^	0.86	2.01	2.873 (6)	176
N6—H6···O6^iv^	0.86	1.99	2.831 (6)	167
N7—H7*B*···O5^iv^	0.86	2.15	2.969 (7)	160
C20—H20···O3^v^	0.98	2.57	3.426 (7)	145
N3—H3*C*···Cl2^vi^	0.86	2.38	3.224 (7)	165
N7—H7*A*···Cl4^vi^	0.86	2.63	3.465 (6)	164
N8—H8*B*···Cl3^vi^	0.86	2.41	3.269 (6)	173
N11—H11*C*···Cl5^vii^	0.86	2.28	3.134 (6)	170
N12—H12*A*···Cl6^vii^	0.86	2.83	3.574 (6)	146
N16—H16*F*···Cl8^vii^	0.86	2.32	3.159 (6)	166
N11—H11*D*···Cl4^viii^	0.86	2.71	3.310 (5)	128
C17—H17*A*···Cl5^viii^	0.97	2.79	3.588 (6)	140
C23—H23*A*···Cl8^ix^	0.97	2.73	3.624 (7)	154
N16—H16*E*···Cl1^ix^	0.86	2.59	3.314 (5)	142
N8—H8*A*···Cl6^x^	0.86	2.69	3.300 (5)	130
N3—H3*D*···Cl7^xi^	0.86	2.70	3.329 (6)	131
N5—H5*D*···O8^xii^	0.90	2.33	3.041 (7)	136
C9—H9*B*···O7^xii^	0.97	2.60	3.460 (7)	148
N13—H13*A*···O4^v^	0.90	2.54	3.081 (7)	120

Symmetry codes: (i) −*x*, *y*+1/2, −*z*+1; (ii) −*x*+2, *y*−1/2, −*z*+2; (iii) −*x*+1, *y*−1/2, −*z*+1; (iv) −*x*+1, *y*+1/2, −*z*+2; (v) *x*−1, *y*, *z*−1; (vi) *x*, *y*+1, *z*; (vii) *x*, *y*−1, *z*; (viii) −*x*+1, *y*−1/2, −*z*+2; (ix) −*x*, *y*−1/2, −*z*+1; (x) −*x*+2, *y*+1/2, −*z*+2; (xi) −*x*+1, *y*+1/2, −*z*+1; (xii) *x*+1, *y*, *z*+1.
